# Bulk and surface recombination properties in thin film semiconductors with different surface treatments from time-resolved photoluminescence measurements

**DOI:** 10.1038/s41598-019-41716-x

**Published:** 2019-03-29

**Authors:** Thomas P. Weiss, Benjamin Bissig, Thomas Feurer, Romain Carron, Stephan Buecheler, Ayodhya N. Tiwari

**Affiliations:** 1 Laboratory for Thin Films and Photovoltaics, Empa–Swiss Federal Laboratories for Materials Science and Technology, Überlandstrasse 129, 8600 Dübendorf, Switzerland; 2 Present Address: Laboratory for Photovoltaics, Physics and Materials Science Research Unit, University of Luxembourg, L-4422 Belvaux, Luxembourg

## Abstract

The knowledge of minority carrier lifetime of a semiconductor is important for the assessment of its quality and design of electronic devices. Time-resolved photoluminescence (TRPL) measurements offer the possibility to extract effective lifetimes in the nanosecond range. However, it is difficult to discriminate between surface and bulk recombination and consequently the bulk properties of the semiconductor cannot be estimated reliably. Here we present an approach to constrain systematically the bulk and surface recombination parameters in semiconducting layers and reduces to finding the roots of a mathematical function. This method disentangles the bulk and surface recombination based on TRPL decay times of samples with different surface preparations. The technique is exemplarily applied to a CuInSe_2_ and a back-graded Cu(In,Ga)Se_2_ compound semiconductor, and upper and lower bounds for the recombination parameters and the mobility are obtained. Sets of calculated parameters are extracted and used as input for simulations of photoluminescence transients, yielding a good match to experimental data and validating the effectiveness of the methodology. A script for the simulation of TRPL transients is provided.

## Introduction

Minority carrier lifetime $\tau$ is generally evaluated by using time-resolved photoluminescence (TRPL) in thin film semiconductors, such as Cu(In,Ga)Se_2_ (CIGS)^1–5^, CdTe^6^ or perovskite^7–10^. However, these photo luminescence (PL) decay times are influenced by surface recombination and hence do not yield the bulk lifetime, which is a desired value indicating the quality of the semiconductor under test. A straightforward extraction of the bulk lifetime from the measured decay of the PL signal is generally not possible since various recombination channels happen simultaneously and are also influenced by diffusion processes^11^. An interpretation of the TRPL decay time as the bulk lifetime leads in turn to an underestimation, which might result in incorrect conclusions. Fitting approaches are also not well suited as several sets of parameters can generally very well reproduce the experimental transients. Several methods were proposed to decouple the effects of diffusion and surface recombination from the bulk recombination. Transient reflectance spectroscopy was proposed to measure the surface recombination as well as the diffusion into the bulk on perovskite single crystals^12^ but can only be applied to free surfaces. On the other hand, passivated surfaces are required to stabilize the interface properties and cannot be measured with the method proposed in^12^. The extraction of charge carriers facilitated by hole or electron transporting layers has also been used as a measure for the bulk lifetime and diffusion coefficient of perovskite absorber layers^9,10^. In that case however, the drift of charge carriers^13^ due to a built-in electric field was not taken into account, leading to an underestimated bulk lifetime and an overestimated diffusion coefficient.

Another method was proposed by Barnard *et al*.^14^ and Kuciauskas *et al*.^15^ that is based on two-photon excited TRPL. With this method carriers are excited deeper inside the bulk of the semiconductor, which decreases the influence of the front surface recombination on the initial decay characteristics of the PL signal^14,15^. Still, the mono-exponential tail decay time only corresponds to an effective lifetime governed by bulk- and surface recombination and not the bulk lifetime of the semiconductor^15^.

Ahrenkiel and Johnston proposed an approach based on resonant-coupled photoconductive decay data in the microsecond range to determine the surface recombination velocity and diffusion constant for Si wafers^16^. A multi-dimensional least square fit is applied to discriminate the obtained parameters. Hempel *et al*.^17^ followed a similar approach by using terahertz spectroscopy, which allows a resolution of 0.1 ps and therefore enables the fitting of the initial decay curve and thus the surface recombination velocity for thin films with carrier dynamics in the sub nanosecond range. However, this methodology cannot be adapted to TRPL data due to a limited temporal resolution^17^. In particular, we show by simulations that a large range of parameters can describe reasonably well the PL decay curves, which limits the use of such a fitting approach.

Staub *et al*. determined an upper limit of the surface recombination velocity assuming that the non-radiative lifetime is solely caused by interface recombination^7^. This approach might be useful for high PL decay times observed for instance in high quality perovskite layers^7^, however, might not yield reasonable limits for other thin film technologies with lower effective lifetimes.

In this paper we present a technique based on the mathematical treatment of PL decay times acquired from compound semiconducting layers with different front and back surface modifications. A model involving surface and Shockley-Read-Hall (SRH) recombination as well as diffusion of excited charge carriers is applied to discriminate these recombination channels. The analysis is carried out on CuInSe_2_ (CIS) and single back-graded Cu(In,Ga)Se_2_ (bg-CIGS) layers used in solar cells^18^ with various surface modifications. These materials were chosen due to their complexity and the possibility to introduce a bandgap grading throughout the semiconducting layer. However, the approach can be applied to other semiconducting material systems as well. Based on the model, upper and lower bounds for the surface recombination velocities are extracted as well as estimates for the bulk properties such as the minority carrier mobility and bulk lifetime. A Matlab script is used to simulate transients based on possible sets of parameters, which reproduce the experimental transients. The source code of this script is available in the Supplementary Information.

The manuscript is structured as follows: section 2 introduces the theoretical background for the analysis of the TRPL decay time based on bulk and surface recombination contributions. Section 3 describes the experimental details for the measurement of TRPL transients as well as the sample preparation. The analysis of the decay times and the discussion of material and interface parameters are given in section 4. Simulated transients using sets of parameters extracted from the mathematical methodology are presented in section 5. The range of valid values for all parameters involved in the underlying model is discussed.

## Theoretical background

### Device model and mathematical solution for a TRPL transient

**Figure 1:**
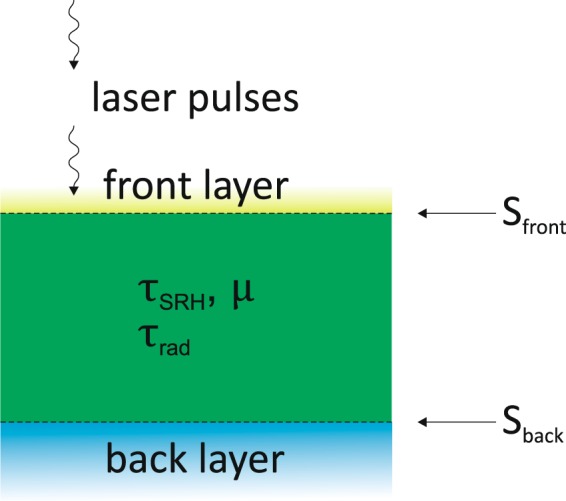
Model for the TRPL interpretation. Parameters describing non radiative recombination are the surface recombination velocities at the front and back side of the absorbers *S*_*front*_ and *S*_*back*_ as well as the bulk lifetime *τ*_*SRH*_ and mobility *μ* of the CIGS absorber. The radiative recombination is taking into account with *τ*_*rad*_ = 1.7 *μs*.

In order to analyze the PL transients the model shown in Fig. 1 is used. It assumes an absorber layer with a homogeneous non-radiative bulk lifetime ${\tau }_{SRH}$ and mobility $\mu$. In particular, the electron and hole mobilities $\mu$ are assumed to be equal and hence no ambipolar transport is considered. Thus, in low injection conditions (as realized in the experiments in the present paper), the minority carrier lifetime is probed, while in high injection conditions the smaller mobility of the two charge carriers is probed^19^. Also ${\tau }_{SRH}$ is equal for electrons and holes, which can be realized by midgap defect states according to ${\tau }_{SRH}=1/{v}_{th}\sigma {N}_{t}$ (under low injection conditions^20^), where ${v}_{th}$ is the thermal velocity of free charge carriers, $\sigma$ the capture cross section for electrons and holes of the midgap defect state and ${N}_{t}$ its total density. In addition, surface recombination at the front and back side of the absorber is included and is described by the recombination velocities ${S}_{front}$ and ${S}_{back}$, respectively.

The radiative recombination rate *R*_*rad*_ is described by

1 $${R}_{rad}=B(({n}_{0}+{\rm{\Delta }}n)\,({p}_{0}+{\rm{\Delta }}n)-{n}_{0}{p}_{0})$$

The equilibrium densities for electrons and holes are denoted by ${n}_{0}$ and ${p}_{0}$, respectively and the excess electron and hole density by ${\rm{\Delta }}n$. For a p-type semiconductor (${p}_{0}\gg {n}_{0}$) and under low injection conditions (${p}_{0}\gg {\rm{\Delta }}n$), Eqn. (1) reduces to

2 $${R}_{rad}=B{p}_{0}{\rm{\Delta }}n=\frac{{\rm{\Delta }}n}{{\tau }_{rad}}$$

with the radiative lifetime ${\tau }_{rad}=1/B{p}_{0}$^21^. For Eqn. (2) it is assumed that $\,{\rm{\Delta }}n={\rm{\Delta }}p$, i.e. without trapping and charge carrier separation. The radiative recombination constant *B* is taken from ref.^20^ and is set to $1.67\times {10}^{-10}\,c{m}^{3}{s}^{-1}$ (see also Table 1). Capacitance voltage measurements were applied to determine $\,{p}_{0}=3.5\times {10}^{15}c{m}^{-3}$ (see Supplementary Information B for more details). Thus, the radiative lifetime evaluates to ${\tau }_{rad}\approx 1.7\,\mu s$ and is kept constant in this study.

**Table 1:** Parameters used for simulating PL transients.

Description	Notation	Unit	Value
rad. rec. coefficient	$B$	$c{m}^{3}{s}^{-1}$	$1.67\times {10}^{-10}$
absorption coefficient	$\alpha$	$\mu {m}^{-1}$	$8.12$
absorber doping	${p}_{0}$	$c{m}^{-3}$	$3.5\times {10}^{15}$
absorber thickness	$d$	$\mu m$	$3.0$
eff. density of states	${N}_{c},\,{N}_{v}$	$c{m}^{-3}$	$2\times {10}^{18}$
temperature	$T$	$K$	$300$

#### Mathematical solution

For a mathematical solution of the PL transient the continuity Eqn. (3) including drift and recombination needs to be solved.

3 $$\frac{\partial {\rm{\Delta }}n}{\partial t}=-\,{R}_{rad}-{R}_{SRH}+\frac{\mu kT}{q}\frac{{\partial }^{2}{\rm{\Delta }}n}{\partial {z}^{2}}+g$$

where $g$ denotes the generation profile of the laser pulse (see Eqn. (13). In the following, the pulse is assumed infinitesimal short, i.e. $exc(t)=\delta (t)$ with ${n}_{\gamma }$ the number of photons per pulse and area. Solutions for other pulse profiles can then be obtained by convolution^11^. Using low injection conditions for the radiative and SRH recombination rate ${R}_{rad}$ and ${R}_{SRH}$, Eqn. (3) reads

4 $$\frac{\partial {\rm{\Delta }}n}{\partial t}=-\,{\rm{\Delta }}n(\frac{1}{{\tau }_{rad}}+\frac{1}{{\tau }_{SRH}})+\frac{\mu kT}{q}\frac{{\partial }^{2}{\rm{\Delta }}n}{\partial {z}^{2}}$$

The solution for Eqn. (3) was calculated (without ${\tau }_{rad}$) using the surface recombination velocities ${S}_{front}$ and ${S}_{back}$ as boundary conditions^11,22^. The solution (including $\,{\tau }_{rad}$ in this case) reads^11,22,23^:

5 $${\rm{\Delta }}n(t,\,z)={A}_{0}+\sum _{k=1}^{\infty }{A}_{k}{U}_{k}(z)\exp (\,-\,{\eta }_{k}t)$$

with

6 $${\eta }_{k}=\frac{1}{{\tau }_{rad}}+\frac{1}{{\tau }_{SRH}}+D{\beta }_{k}^{2}$$

and

7 $$\tan \,{\beta }_{k}d=-\frac{D({S}_{back}+{S}_{front}){\beta }_{k}}{{S}_{back}{S}_{front}-{D}^{2}{\beta }_{k}^{2}}$$

Here $D=\mu kT/q$ denotes the diffusion constant and $d$ the thickness of the semiconductor layer. The functions ${U}_{k}(z)$ and prefactors ${A}_{k}$ are dependent of the surface recombination and are given by^11,23^

8 $${U}_{k}(z)=cos{\beta }_{k}z+\frac{{S}_{f}}{D{\beta }_{k}}sin{\beta }_{k}z$$

9 $${A}_{k}=\alpha {n}_{\gamma }\frac{{\int }_{0}^{d}dz\,\exp -\alpha z{U}_{k}(z)}{{\int }_{0}^{d}dz\,{U}_{k}^{2}(z)}$$

Equation(7) is solved numerically for ${\beta }_{k}$ with $0 < {\beta }_{1} < {\beta }_{2} < \ldots$ . As the ${\beta }_{k}$ coefficients are strictly increasing with *k*, the contribution to ${\rm{\Delta }}n(t,z)$ of the summed terms at later times is decreasing exponentially due to the term $\exp (-{\eta }_{k}^{2}t)$ in Eqn. (5). Consequently, the decay at long times of a measured transient can be described by using only the first term with $k=1$, which describes the recombination at the surfaces and in the bulk^11,24^. Note that ${A}_{0}=0$ if either of the surface recombination velocities is non-zero^23^. Consequently, at late times Eqn. (5) reduces to

10 $$\Delta n(t,\,z)={A}_{1}{U}_{1}(z)\exp (-[\frac{1}{{\tau }_{rad}}+\frac{1}{{\tau }_{SRH}}+D{\beta }_{1}^{2}]t)$$

Late times refers to the time until the injected carriers diffused into the semiconducting layer and are in quasi equilibrium, i.e. the surface recombination constant is not dependent on time anymore. Maiberg *et al*. showed that diffusion takes place within a few ns for a mobility $> 10\,c{m}^{2}{V}^{-1}{s}^{-1}$ and a layer thickness of $3\,\mu m$. Experimentally, this can be observed by a single exponential decay.

As ${A}_{1}$ and ${U}_{1}(z)$ are time independent in Eqn. (10), the effective lifetime ${\tau }_{eff}$ can be written as

11 $$\frac{1}{{\tau }_{eff}}=\frac{1}{{\tau }_{rad}}+\frac{1}{{\tau }_{SRH}}+\frac{1}{{\tau }_{surf}}$$

with

12 $$\frac{1}{{\tau }_{surf}}=D{\beta }_{1}^{2}$$

It is stressed that the surface term ${\tau }_{surf}$ includes the surface recombination at the front- and the backside via Eqn. (7) as well as the mobility, which is included in the diffusion constant. Therefore the effective tail decay time ${\tau }_{eff}$ can be expressed as a function of 4 free parameters: ${S}_{front},\,{S}_{back},\,\mu ,\,{\tau }_{SRH}$.

In section 4 the expression (11) is used to calculate the effective lifetime based on these four parameters. Then the comparison to experimental PL decay times allows deducing boundary values for these 4 parameters. It is pointed out that good analytical approximations for Eqn. (7) exist in the two special cases: ${S}_{front}={S}_{back}=S$ or that either of the recombination velocities is zero^11,22^. However, in the general case of ${S}_{back}\ne {S}_{front}\ne 0$, which is used in the present paper, Eqn.(7) needs to be solved numerically.

Recently, trapping of minority carriers has also been proposed to influence the measured decay tail time^25–27^, which was mainly motivated by a strong temperature dependence of the decay tail time^26^. While trapping was included into the model presented in^27^, no temperature dependent transients were measured. Redinger *et al*. measured temperature dependent transients in air and in N_2_ environment and showed that the strong temperature dependence can result from a degradation of the front surface^28^. For the CIS and the bg-CIGS absorbers investigated in this study the TRPL curves do not show a strong temperature dependence and thus might be explained solely by the temperature dependence of the Shockley-Read-Hall recombination rate (see Supplementary Fig. 3). It is noted that this argument alone is not sufficient to exclude an influence of trapping. However, due to simplicity, trapping is not included in the analysis presented in this paper.

### Simulations of time-resolved photoluminescence transients

If not mentioned otherwise, 1D simulations of PL transients were carried out using a Matlab script, which is provided in the supporting information. The script solves the drift-diffusion equation as well as the continuity equation for electrons and holes. Bulk defects are implemented using SRH statistics and surface recombination by surface recombination velocities. The Poisson equation is not solved and hence ambipolar transport is not taken into account. Further details concerning the physics of the Matlab script are given in Supplementary Information A.

The conduction band grading for one of the semiconductor under test (see section 3.2) is implemented on each mesh point by a potential energy for the electrons. Material parameters used for the simulations are listed in Table 1. Other parameters, such as bulk lifetime, surface recombination velocities and mobilities are estimated and discussed in the main text.

In order to verify the results obtained by the Matlab script a number of transients with relevant parameter variations were simulated using Sentaurus TCAD, see supporting information A. For TCAD simulations the graded conduction band was implemented by splitting the semiconductor region into 40 sub-regions and by linearly interpolation between these regions subsequently.

#### Optical generation

Experimentally, free electron-hole pairs are generated by a pulsed laser, as described in the experimental section 3.1. For the simulations the absorption and hence the generation is described by Beer-Lambert law according to

13 $$g(z,\,t)\propto {n}_{\gamma }exc(t)\alpha \exp (-\alpha z)$$

with the absorption coefficient $\alpha =\,8.12\,\mu {m}^{-1}$ at 639 nm, which was determined by in-house measurements of CIS layers^29^. The normalized function $exc(t)$ describes the shape of the laser pulse, which is used for simulated transients (section 5), and the $\alpha$ factor normalizes the exponential term. The total number of injected electron-hole pairs per pulse and per area ${n}_{\gamma }$ is estimated from the laser power and excitation spot area.

## Experimental

### TRPL measurements

For TRPL measurements the sample is excited with a 639 nm laser on a spot of roughly 50 $\mu m$ diameter with a pulse FWHM of around 100 ps. The PL emission is collected by an optical system with numerical aperture of 0.22 and detected by a Peltier cooled InGaAs photomultiplier tube. A monochromator in the detection chain is set to 0^th^ order, i.e. the PL transients were spectrally integrated. The excitation density was estimated by measuring the current of a Si reference cell for the different laser intensity settings used in this study.

### Sample preparation

The samples were grown on Mo coated glass substrates by a multistage co-evaporation process as described in ref.^18^ (samples CIS and BG2 therein). For the CuInSe_2_ (CIS) sample only In was supplied as a group-III element during the growth, resulting in a constant bandgap through the depth of the absorber. For the single graded Cu(In,Ga)Se_2_ (bg-CIGS) sample, Ga was supplied within the first stage of the deposition process, realizing a Ga gradient only towards the back contact. The front side of the bg-CIGS layer has a bandgap of around 1 eV similar to the pure CuInSe_2_ absorber and hence similar absorption characteristics^18^. The [Ga]/([Ga] + [In]) (GGI) grading profile as measured by secondary ion mass spectrometry (SIMS) of the bg-CIGS absorber investigated here is shown in Supplementary Fig. 1. (black line). The layer shows a GGI at the back of 0.28. For simulated transients the GGI grading is implemented as a conduction band grading as detailed in section 2.2. Parts of the absorber layers were completed into solar cell devices with a stack of CdS/ZnO/Al:ZnO and a Ni/Al grid (see ref.^18^ for details).

**Figure 2:**
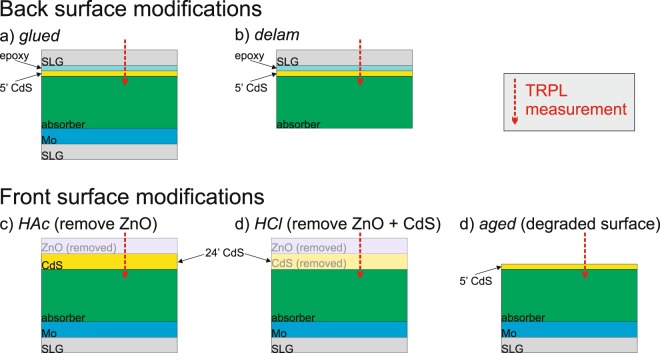
Sample modifications for measuring the same absorber with different surface conditions.

TRPL measurements were carried out on the absorbers with various surface treatments, and not on full devices to exclude severe charge separation effects^30^. These surface treatments were applied to modify the front or the back surface of the absorber and consequently the surface recombination velocities. Figure 2 displays the various configurations and modifications for the front and back surfaces. These configurations can be described as follows:

i. *glued*: a thin CdS layer was grown by a 5 minute chemical bath deposition (CBD) process to stabilize and passivate the front surface of the absorber layer^31,32^. The deposition time was kept short to limit the CdS thickness and to avoid the formation of an electric field due to the formation of a pn junction^33^ (see also the configuration *HAc*). After CdS deposition a glass substrate was glued on top with an approximately 30 $\mu m$ thick transparent epoxy layer. It was checked that the epoxy layer does not emit parasitic luminescence during a TRPL measurement. For this configuration the absorber back surface remains in contact to the Mo/glass substrate.
ii. *delam*: The sample is prepared as described for the *glued* configuration. However, the sample is subsequently detached from the Mo/glass substrate. The back side of the absorber is not in contact to the Mo layer anymore, but exposed to air.
iii. *HAc*: Starting from a finished device the ZnO was removed by 1 min etching (sonication) in 5% acetic acid (HAc) leaving only approximately 50 nm (24 min CBD) of CdS (see Supplementary Fig. 2). The back side of the absorber is in contact to the Mo/glass substrate. It is noted that TRPL measurements of the device after removal of the window layer are very similar to those of an absorber directly after the deposition of a thick CdS layer (not shown).
iv. *HCl*: Starting from a finished device the ZnO and the CdS layers were removed by 1 min etching (sonication) in 5% hydrochloric acid (HCl) leaving the bare absorber surface exposed (see Supplementary Fig. 2). The back surface is in contact to the Mo/glass substrate. For the CIS sample the HCl etch led to uncontrolled flaking of the absorber. Thus, this configuration could only be applied for the bg-CIGS absorber.
v. *aged*: same as i) but without gluing a glass substrate onto the front side. The sample with a thin CdS layer did not show any degradation in a TRPL measurement within a few days. However, after storing the sample in ambient conditions for approximately 3 months a degradation was observed in terms of a faster PL decay. In the subsequent analysis the configuration *aged* denotes the condition after the 3 months storage. It is noted that the configuration *glued* did not show any signs of degradation.

## Results and Discussion

**Figure 3:**
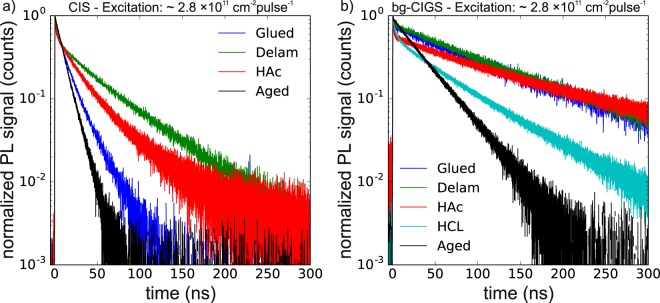
Normalized PL transients of the CIS and the back graded CIGS absorber. Various surface modifications are applied demonstrating the effect of the surface recombination velocity.

Figure 3 shows normalized PL transients measured on the CIS and the bg-CIGS absorber for the surface modifications described in section 3.2. In general, the transients show a small initial non-exponential decay (during the first few ns after the excitation) and a single-exponential decay after approximately 20 ns. The focus of the subsequent analysis is the single-exponential decay at late times (after 20 ns). In this regime it is assumed that the system can be described by low injection conditions and that the PL decay time can be described by Eqn. (11), i.e. by a single exponential decay with effective lifetime ${\tau }_{eff}$. Low injection conditions in this regime are indicated by the single exponential behavior. In addition, assuming that the carriers have homogenized in the $3\,\mu m$ thick absorber (in the single exponential region) results in an excess carrier density of $2.8\times {10}^{11}\,c{m}^{-2}/3\,\mu m\approx 1\times {10}^{15}\,c{m}^{-3}$, which is below the doping density of $3.5\times {10}^{15}c{m}^{-3}$. In the following sections the lifetimes were extracted consistently between 20 ns and 80 ns to prevent any influence of possible high-injection conditions and residual background. As the modification of the surfaces is not expected to influence the bulk properties, the changes in the decay time for different sample configurations can be assigned to the front and back surface recombination terms given by $\,{\tau }_{surf}^{-1}=D{\beta }_{1}^{2}$. In the following, the results obtained for the CIS (section 4.1) and the bg-CIGS (section 4.2) absorbers are investigated separately. Subsequently, the obtained recombination parameters are discussed in section 4.3 and compared to simulated transients (section 5).

### Surface recombination analysis for the CIS absorber

Table 2 reports the experimental decay times, which span from 11 ns (*aged*) to 48 ns (*delam*) for the CIS absorber. An important observation is that the decay time in the *delam* configuration is strongly increased compared to the *glued* configuration (see also Fig. 3a, green and blue curve, respectively). The only difference is the back contact and hence the back surface recombination, which for the *glued* configuration (in contact with the Mo/glass substrate) is much stronger. For the following analysis, the surface recombination velocity of the CIS/Mo interface is set to $\,{S}_{back,Mo}=1\times {10}^{6}\,cm{s}^{-1}$ (see Supplementary Information B for further discussion). It is noted that similar results are obtained with a weaker $\,{S}_{back,Mo}=1\times {10}^{5}\,cm{s}^{-1}$ and a stronger ${S}_{back,Mo}=1\times {10}^{7}\,cm{s}^{-1}$ back surface recombination velocity (not shown).

**Table 2:** Decay times of measured PL decay curves shown in Fig. 3.

Measured decay times in ns					
	*glued*	*delam*	*HAc*	*HCl*	*aged*
CIS	17	48	30	—	11
bg-CIGS	98	105	118	57	34

The decay times were extracted from a single exponential fit in the range 20 ns to 80 ns.

In the following, a systematic approach is described to confine the value range of the remaining parameters based only on the effective decay times presented in Table 2. As discussed in section 2.1.1 each of these effective decay times is determined by a set of parameters $({\tau }_{bulk},\mu ,{S}_{front},{S}_{back})$ through Eqn. (11).

**Figure 4:**
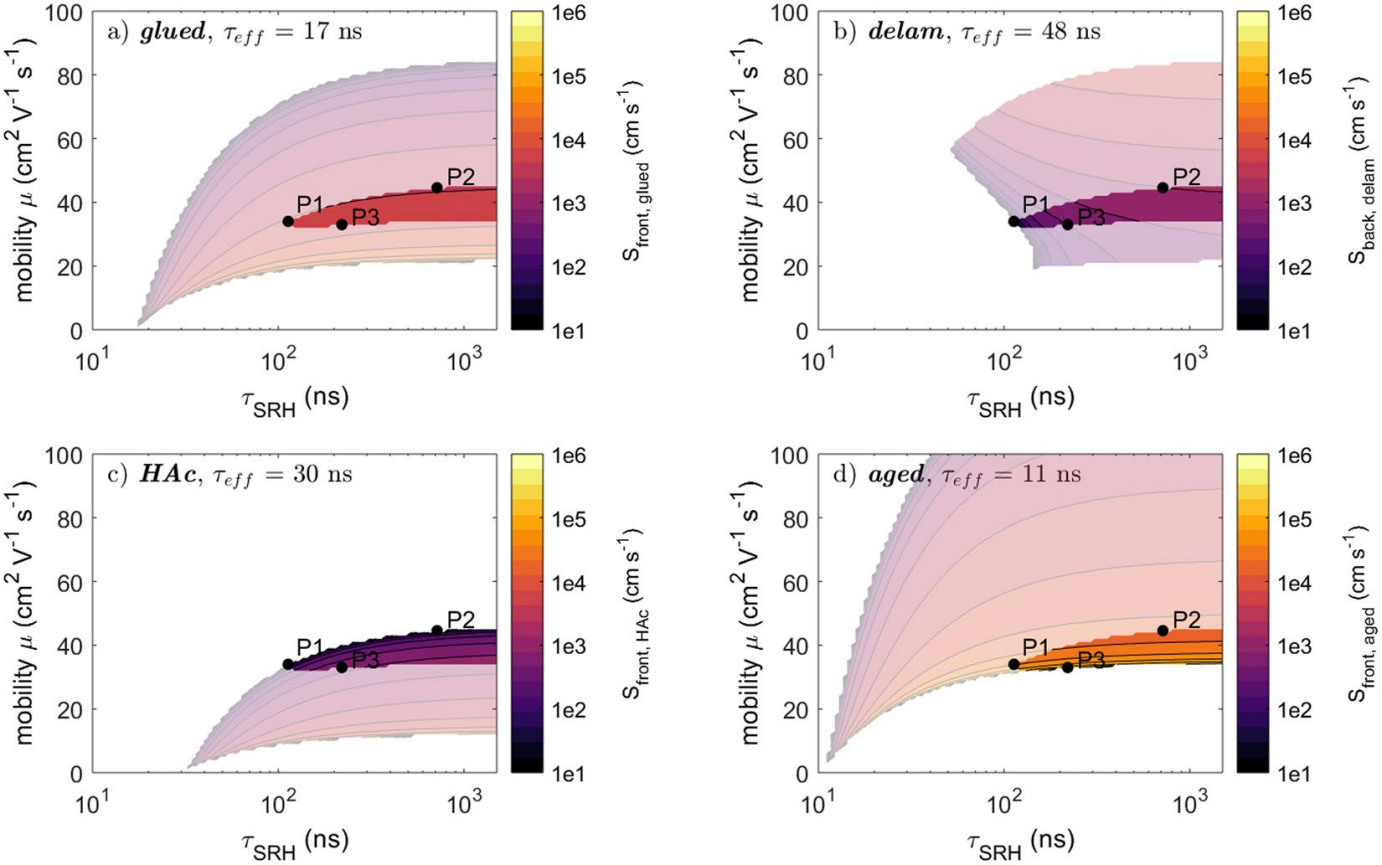
Estimation of the valid parameter values of *τ*_*SRH*_, *μ* and the unknown surface recombination velocity of the corresponding sample configuration according to Eqn. (14) for the CIS absorber. The pale color maps in the background indicate the parameters satisfying Eqn. (14) when only a single measurement for the indicated configuration is taken into account. The bright (and smaller) color map is the intersection of all individual color maps. These bright maps take also the restrictions for *τ*_*SRH*_ and *μ* into account, which are given by the measurements of the other configurations.

To explore the parameter space a numerical solution has been calculated to the equation

14 $${\tau }_{exp,i}={\tau }_{eff}({\tau }_{bulk},\mu ,{S}_{front,i},{S}_{back,i})$$

where ${\tau }_{exp,i}$ denotes the experimentally measured lifetime for the configuration *i* (*glued*, *delam*, *HAc*, *aged*) reported in Table 2. For the configurations in contact with the Mo substrate the back surface recombination velocity was set to the fixed value ${S}_{back,Mo}=1\times {10}^{6}cm{s}^{-1}$ as described above. For the *delam* configuration the front surface is the same as for the *glued* configuration and therefore ${S}_{front,delam}={S}_{front,glued}$. Consequently, for each configuration the parameter space to Eqn. (14) is reduced down to 3 free variables. By imposing values to the $({\tau }_{bulk},\,\mu )$ parameters, a solution may be found for the surface recombination velocity such that Eqn. (14) is fulfilled. Figure 4 presents the solutions to the surface recombination velocity as a function of the two absorber parameters ${\tau }_{bulk}$ and $\mu$, for each sample configuration. A white background indicates that no solution exists for that particular combination of (${\tau }_{bulk},\mu$). The pale colors represent combinations of (${\tau }_{bulk},\mu$) were solutions were found based on Eqn. (14), for each configuration separately. Thus, each configuration results in different restrictions to the parameter space. As the same absorber is used for all surface configurations, the values for ${\tau }_{bulk}$ and $\mu$ are expected to be the same and independent of the surface configuration. Therefore, valid (${\tau }_{bulk},\mu$) values must allow solutions for each and every sample configuration. These regions are obtained by the intersection of the valid parameter space of each individual configuration and are displayed as bright regions in Fig. 4. These maps show that the parameters governing the recombination can effectively be confined by measurements on different sample configurations (*glued*, *delam*, *HAc*, *aged*). Boundary values for each recombination parameter are then extracted by minimum and maximum value in each (intersected) map and are listed in Table 3. The mobility is confined between 32 and 45 $c{m}^{2}{V}^{-1}{s}^{-1}$ and the bulk lifetime takes values above 117 ns. This value is significantly larger than the highest measured decay time (48 ns for the *delam* configuration) and shows a clear influence of the surface recombination on experimental data.

**Table 3:** Boundary values for the surface recombination velocities and the absorber properties based on the mathematical analysis presented in sections 4.1 (CIS) and 4.2 (bg-CIGS).

		CIS		bg-CIGS	
		lower limit	upper limit	lower limit	upper limit
${S}_{front,glued}$	*cms*^−1^	$5.3\times {10}^{3}$	$9.1\times {10}^{3}$	$3.0\times {10}^{2}$	$1.8\times {10}^{3}$
${S}_{front,HAc}$	*cms*^−1^	0	$1.4\times {10}^{3}$	$4.4\times {10}^{1}$	$1.4\times {10}^{3}$
${S}_{front,aged}$	*cms*^−1^	$2.2\times {10}^{4}$	$\infty$	$3.2\times {10}^{3}$	$\infty$
${S}_{front,HCl}$	*cms*^−1^	n/a		$1.4\times {10}^{3}$	$4.7\times {10}^{3}$
${S}_{back,delam}$	*cms*^−1^	0	$1.9\times {10}^{3}$	n/a	
${\tau }_{bulk}$	*ns*	117	$\infty$	132	∞
$\mu$	*cm*^2^*V*^−1^*s*^−1^	32	45	8.3	∞ (*)

For the calculations of the CIS absorber a surface recombination velocity at the Mo interface was chosen to be *S*_*back*_,_*Mo*_ = 10^6^ *cms*^−1^. (*) Simulations of transients could provide an upper limit to the mobility *μ* in the bg-CIGS absorber.

In section 4.3 three parameter sets P1, P2 and P3 obtained from this approach will be used as input for simulated transients. It will be shown that the parameters indeed result in correct decay times and can describe the experimental transients with a reasonable match.

### Surface recombination analysis for the bg-CIGS absorber

**Figure 5:**
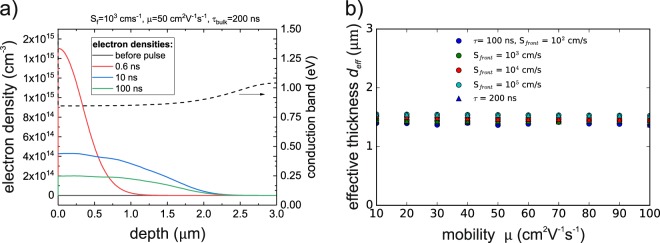
(**a**) TCAD simulations of the electron density within the back graded absorber bg-CIGS for different times after the excitation pulse. The back grading results in a decreased electron density towards the back confining the electrons within an effective depth $\,{{d}}_{{eff}}$. (**b**) Calculated effective thickness for the back graded CIGS absorber according to Equations (7) and (11) with the same parameters *τ*_*bulk*_, *S*_*front*_, *μ* as used by the TCAD simulations.

Figure 3b shows the experimental transients for the configurations used for the bg-CIGS absorber and Table 2 reports the extracted decay times. Compared to the CIS absorber the transients for the *glued* and the *delam* configuration are very similar, demonstrating that the Ga back grading (increase of GGI by 0.28) efficiently prevents the recombination at the back contact. Simulations support this assumption (presented in Supplementary Fig. 4), which show that a conduction band increase of 193 meV towards the back contact (equivalent to a GGI at the back contact of 0.28) yields transients independent of the back surface recombination velocity. The recombination at the back contact is prevented by a very low electron density in this high bandgap region, while the electrons are concentrated in the low bandgap region (Fig. 5a). This effect is accounted for in the solution of Eqn. (11) by modeling the bg-CIGS absorber as an ungraded layer with a thinner effective thickness ${d}_{eff}$ and a low back surface recombination velocity. In order to set a reasonable ${d}_{eff}$ value, TCAD transients were simulated implementing the grading shown in Supplementary Fig. 1 using a few sets of input parameters ${S}_{front},\,{S}_{back},\,\mu ,\,{\tau }_{bulk}$. The simulated decay times were used in the model of Eqn. (11): by imposing the same ${S}_{front},\,\mu ,\,{\tau }_{bulk}$ parameters and ${S}_{back}=0\,cm{s}^{-1}$ a solution of the effective thickness ${d}_{eff}$ is calculated and presented in Fig. 5a. The solution of ${d}_{eff}$ is consistently between 1.4 and 1.6 $\mu m$ and matches roughly the absorber depth, where the increase in the conduction band corresponds to the thermal energy $\,{k}_{b}T$. Thus, for the ongoing analysis of the bg-CIGS absorber a value of ${d}_{eff}=1.5\,\mu m$ was used and ${S}_{back}$ was set to $1\,cm{s}^{-1}$, which represents a passivated back contact.

**Figure 6:**
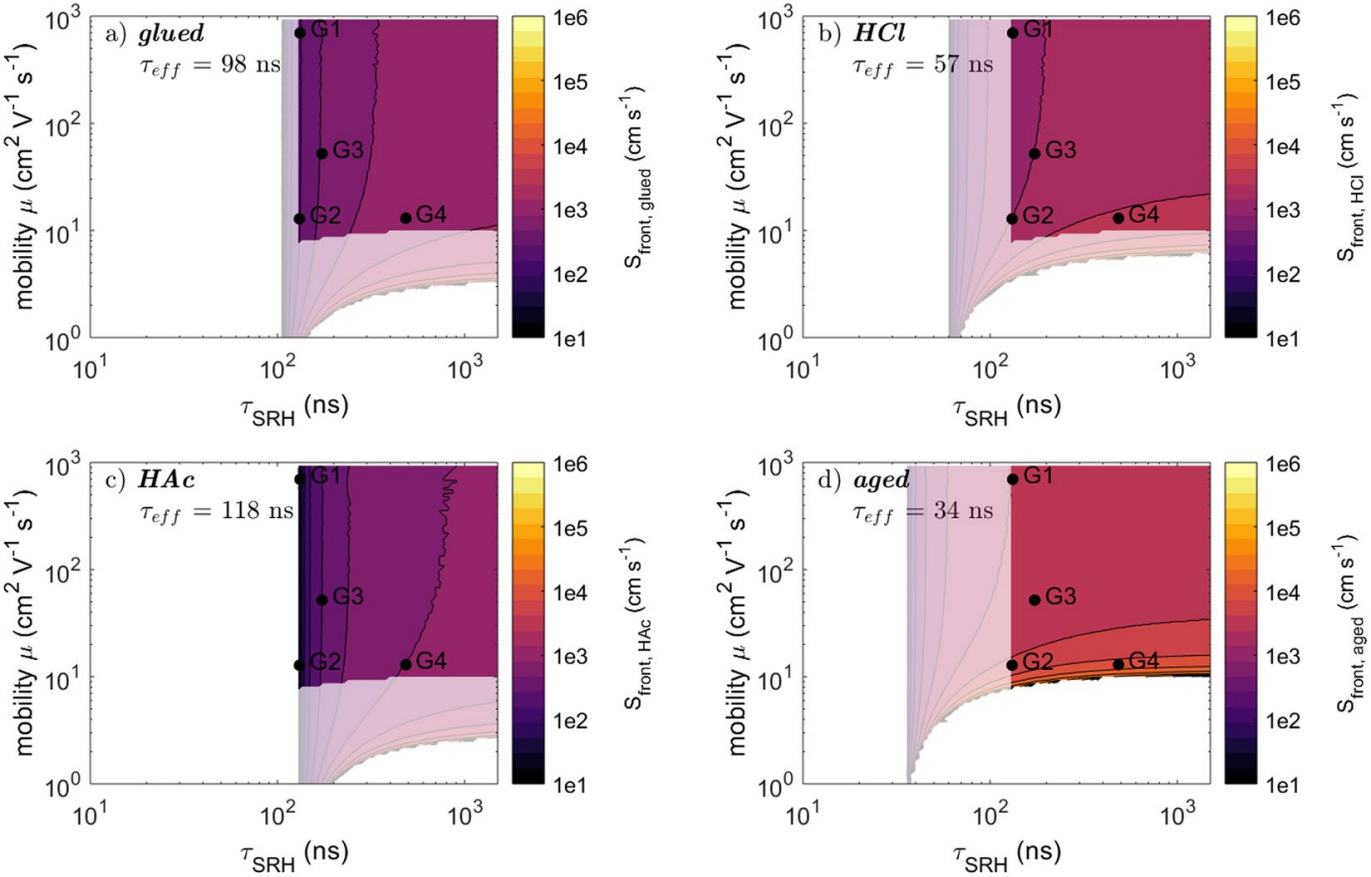
Solutions of the parameters for *τ*_*bulk*_ and *μ* and the surface recombination velocities for the bg-CIGS absorber based on Eqn. (14). The pale areas indicate the solution when taking into account only one decay time for the corresponding sample configuration. Bright areas indicate solutions considering the solutions for all configurations.

Figure 6 shows the valid parameters based on the numerical analysis as similarly carried out for the CIS sample in section 4.1. No map is shown for the *delam* configuration as it is similar to the *glued* case due to similar PL decay times (see Fig. 3b and Table 2). Each map shows the solutions obtained from each individual measurement/configuration (pale areas) as well as the combined solutions (bright areas). The upper and lower bounds are presented in Table 3. The estimates of the surface recombination velocities are comparable to these of the CIS absorber. However, no upper bound of the mobility $\mu$ could be identified for the bg-CIGS absorber, which may take large values. In contrast, the mobility in the CIS absorber was quite well confined. The reason for that will be discussed in section 4.3.

### Discussion and comparison to simulated transients

Table 3 compiles the boundary values estimated from the mathematical analysis of the decay times, for both CIS and bg-CIGS absorbers. While there is a small difference for ${S}_{front,glued}$, there is a good match for the two recombination velocities ${S}_{front,HAc}$ and ${S}_{front,aged}$. Concerning the bulk properties for the CIS absorber the mobility could be confined between 32–45 $c{m}^{2}{V}^{-1}{s}^{-1}$ and a lower bound of 117 ns for the SRH lifetime ${\tau }_{SRH}$ was determined. It is stressed that this lower bound of ${\tau }_{SRH}$ is much larger than any measured PL decay time demonstrating the influence of surface recombination. For the bg-CIGS absorber only a lower limit of 8.3 $c{m}^{2}{V}^{-1}{s}^{-1}$ is identified for the mobility but no upper limit could be deduced. The reason is that for the bg-CIGS absorber no configuration exists with a high enough surface recombination velocity (the back surface is passivated due to the GGI grading). Thus, the parameters (${\tau }_{SRH},\mu ,{S}_{front}$) can always be chosen such that the surface recombination rate is limited by the surface recombination velocity and not by the mobility of the charge carriers, which have to diffuse to the recombinative surfaces. Ahrenkiel^22^ gives approximate analytical expressions for ${\tau }_{surf}$ in the limiting cases of small and high values for $y=\frac{Sd}{D}$ (with $S={S}_{front}={S}_{back}$ for simplicity). For small values of y (i.e. small surface recombination velocity compared to the mobility) the approximation using $\tan \,{\beta }_{k}d={\beta }_{k}d$ can be applied to Eqn. (7) and it is found^22^

15 $${\tau }_{surf}\approx \frac{d}{2S}$$

Clearly, the surface recombination is not dependent on $\mu$, which in turn cannot be confined. In contrast to the bg-CIGS absorber, the CIS absorber exhibits a highly recombinative back contact due to the Mo and the absence of a bandgap gradient. Looking at the decay time and the parameter map in Fig. 4 for the *HAc* configuration, the mobility cannot be increased arbitrarily since for high ${S}_{back}$ (${S}_{back,Mo}={10}^{6}cm{s}^{-1}$) the surface recombination is mobility limited by diffusion to the recombinative surfaces resulting in^22^

16 $${\tau }_{surf}\approx \frac{{d}^{2}}{{D}^{2}{\pi }^{2}}$$

These limiting cases show that a measurement with a highly recombinative surface is necessary for confining the mobility towards large values.

An upper limit for the SRH lifetime ${\tau }_{SRH}$ could neither be deduced for the CIS nor for the bg-CIGS absorber, since the parameters can always be chosen in a way that the effective decay time is limited by surface recombination.

Due to the large spread in (valid) parameter values for ${\tau }_{SRH}$ and $\mu$, it cannot be conclusively evaluated if the difference in the measured decay times (bg-CIGS and CIS absorber) is solely due to a passivated back contact for the bg-CIGS absorber, or if also the bulk properties ${\tau }_{bulk}$ and $\mu$ are improved for the bg-CIGS device.

### Simulation of Transients

**Figure 7:**
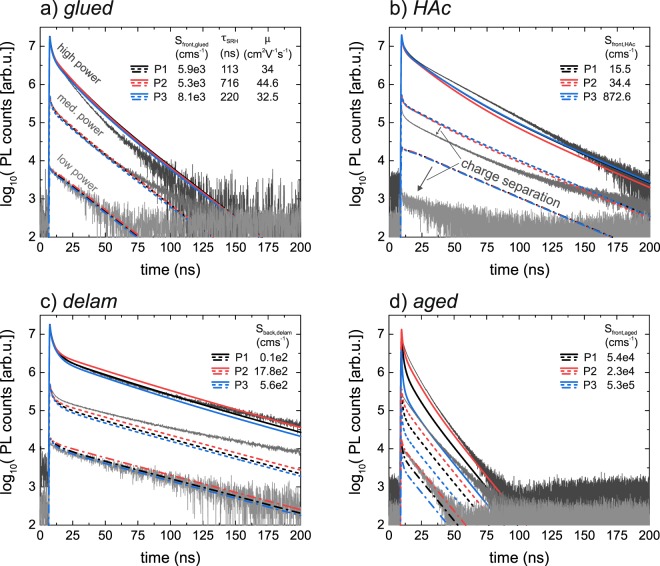
Simulated (colored) and measured (black) transients based on three parameter sets (P1, P2 and P3) deduced by the mathematical considerations in section 4.1. See also Fig. 4 for details of P1, P2 and P3.

**Figure 8:**
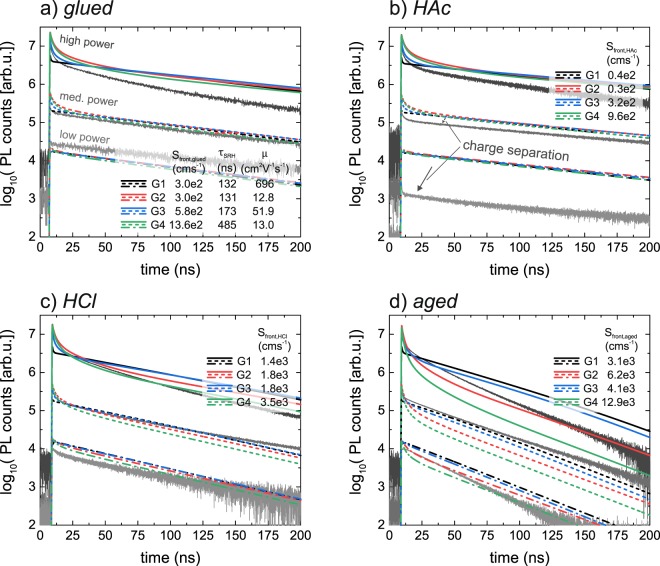
Simulated (colored) and measured (black) transients of the bg-CIGS absorber for the parameter sets G1-G4 as presented in Fig. 6. G1 and G3 are the two sets with rather high mobilities, while G2 and G4 have low *μ* but span a large range of *τ*_*SRH*_.

In the following, simulated TRPL transients confirm the validity of the mathematical approach, and are used to evaluate if more information can be extracted from the measurements. The transients are simulated using the Matlab script for the parameter sets annotated in Fig. 4 (CIS absorber) and Fig. 6 (bg-CIGS absorber) and are shown in Figs 7,8, respectively. Estimations of the experimentally injected charge carriers are implemented, and the transients are scaled (same factor for all transients) to account for the collection efficiency of the optical system. For all configurations, the injection levels were set to $6.4\times {10}^{10}\,c{m}^{-2}puls{e}^{-1}$ (low power), $5.3\times {10}^{11}\,c{m}^{-2}puls{e}^{-1}$ (med. power) and $3.6\times {10}^{12}\,c{m}^{-2}puls{e}^{-1}$ (high power). Except for the *HAc* configuration (see discussion below), a good match for the PL yield is found.

By comparison of the simulated transients with the experimental transients at medium power a good match between the decay times (slopes of the transients) between 20 and 80 ns (time range used as input for the mathematical analysis, see Table 2) is observed. Consequently, the mathematical analysis of the decay times is indeed well suited to determine reasonable device parameters. In particular, the approach of defining an effective thickness *d*_*eff*_ (for a certain back grading) as described in section 4.2 works reasonably well.

Concerning the transients for the CIS absorber a good match for all injection levels is found for the *glued* (Fig. 7a) and the *delam* (Fig. 7c) configuration; also the initial non-exponential decay is well reproduced. Hence, no further confinement of ${\tau }_{SRH}$ and $\mu$ values can be made by taking additionally the initial decay into account, despite the fact that ${\tau }_{SRH}$ ranges between 117 ns and 716 ns and $\mu$ between 32 cm^2^V^−1^s^−1^ and 45 cm^2^V^−1^s^−1^ for the simulated transients shown in Fig. 7a,c. In particular, this shows that fitting a transient can become problematic as similar transients are obtained for large deviations of the recombination parameters (in this case only three parameters:$\,{S}_{front},\,\mu ,{\tau }_{SRH}$).

For the *HAc* configuration the decay times as well as the shape of the initial decay are also well reproduced. However, the experimental maximal PL yield is strongly reduced at low power levels compared to the simulations. This might hint to the fact that the ~50 nm thick CdS layer after the HAc etch introduces a small electric field, which separates the injected charge carriers (already during the pulse) and thus quenches the PL yield^33^. The simulated transients for the *aged* configuration deviate slightly and might be explained that the aged surface cannot be solely described by a surface recombination velocity but probably also surface charge needs to be taken into account. Despite that, the parameter set for P3 with a rather high surface recombination velocity of ${S}_{front,aged}=\,5.2\times {10}^{5}\,{{\rm{cms}}}^{-1}$ shows the strongest deviation in the shape of the initial decay and thus might be excluded. Still, this cannot be used to further confine values of ${\tau }_{SRH}$ or $\mu$.

The simulated transients for the bg-CIGS absorber (Fig. 8) can be analyzed similarly. The simulated decays give a good match with the experimental transients for the configurations *glued* (Fig. 8a) and *HCl* (Fig. 8c) except for high power levels, where experimentally a reduced decay time is observed as compared to lower power levels. Also the shape of the initial decay is well reproduced except for the parameter set G1 with $\mu =696\,c{m}^{2}{V}^{-1}{s}^{-1}$. Due to the high mobility the carriers homogenize rather quickly. Consequently, the non-exponential decay caused by bi-molecular recombination and diffusion is observed for considerably shorter times (compared to the other three parameter sets with lower mobilities). Thus, it can be concluded that the mobilities cannot take arbitrarily high values as otherwise the initial decay is not pronounced enough. The initial decay for the parameter set with $\mu =50\,c{m}^{2}{V}^{-1}{s}^{-1}$ still looks reasonable and thus cannot be excluded. To establish a better confinement for the mobility a more detailed analysis for the bg-CIGS absorbers needs to be carried out in terms of the initial decay characteristics, which is however not subject of this paper. The transients for the *HAc* configuration show a similar behavior as for the CIS absorber, which is a strongly reduced PL yield at low power levels compared to the simulated transients. Again, this might be caused by an electric field present due to the CdS buffer layer. The *aged* transients also deviate slightly as for the CIS case indicating a similar surface modification (i.e. possibly additional surface charges).

It is worth noting that ${S}_{front,HAc} < {S}_{front,HCl}$, with ${S}_{front,HAc} < 1.4\times {10}^{3}cm{s}^{-1}$, which shows the passivating effect of the CdS buffer layer. Further, it is worth noting that an electric field potentially caused by the CdS layer (for the *HAc* configuration) leads to an increased recombination at the front surface due to the attraction of the minority carriers towards the junction. In that case, the upper bound for the real front surface recombination velocity ${S}_{front,HAc}$ is even lower.

With these simulations we demonstrate that the mathematical approach is an efficient method to systematically explore the large parameter space and provide numerical bounds to recombination parameters. In our case, only marginal information could be gained from the more elaborate transient simulations.

## Conclusions

An analysis method is presented to estimate the surface recombination velocities as well as charge carrier mobility and SRH lifetime values based on TRPL measurements. For this purpose, the surface recombination velocities are tuned by applying various surface modifications. This enables to explore systematically the parameter space, to disentangle the contributions of surface and bulk recombination and to determine lower and/or upper bounds of the parameters.

The mathematical analysis was carried out on a CIS and a single back-graded CIGS absorber. Table 3 presents an overview of the bounds for the parameters governing the recombination in these systems. In particular, a surface recombination velocity below $1.4\times {10}^{3}cm{s}^{-1}$ was found for the CIS/CdS interface, which is an important parameter for device simulations. For the CIS absorber the mobility was deduced between $32\,-\,45\,c{m}^{2}{V}^{-1}{s}^{-1}$. For the bg-CIGS absorber the confinement of mobility values was not as good, as no surface configuration could cause sufficiently large surface recombination to be mobility limited. For the bulk lifetime ${\tau }_{SRH}$ a lower limit of ${\tau }_{SRH} > 117\,ns$ was determined for the CIS absorber, indicating relatively good bulk properties even though the highest measured decay time was only 48 ns.

Transients were simulated using a Matlab script with valid sets of device parameters deduced from the mathematical analysis as input. Taking the shape of the initial decay into account, high mobilities around $700\,c{m}^{2}{V}^{-1}{s}^{-1}$ in the bg-CIGS device could be excluded. However, no further confinement of the bulk lifetimes could be achieved based on the simulations of transients compared to the mathematical model. Nevertheless, a good match of the decay tail times is found showing the validity and effectiveness of the mathematical approach. Even for a large range of parameters, the initial decay is generally well reproduced, demonstrating that fitting procedures for the determination of the recombination parameters is problematic. In case of a fitting procedure it is thus recommended to use various excitation wavelengths to vary the shape of the initial decay as for instance applied in^17^.

## Supplementary information

Supplementary Information

Dataset 1

## Data Availability

The datasets generated during and/or analyzed during the current study are available from the corresponding author on reasonable request.
