# Supplementary material for: Bulk and surface recombination properties in thin film semiconductors with different surface treatments from time-resolved photoluminescence measurements
Source: Sci Rep. 2019 Mar 29;9:5385. doi: 10.1038/s41598-019-41716-x (PMC6440953; doi:10.1038/s41598-019-41716-x)
Supplement: Supplementary file 1 — Supplementary Information [file 41598_2019_41716_MOESM1_ESM.pdf]

# Bulk and surface recombination properties in thin film semiconductors with different surface treatments from time-resolved photoluminescence measurements

Thomas P. Weiss[+,1,2], Benjamin Bissig[+,1], Thomas Feurer[1], Romain Carron[1], Stephan Buecheler[*,1], Ayodhya N. Tiwari[1]

*[1]Laboratory for Thin Films and Photovoltaics, Empa – Swiss Federal Laboratories for Materials Science and Technology, Überlandstrasse 129, 8600 Dübendorf.*

*[2]Current Address: Laboratory for Photovoltaics, Physics and Materials Science Research Unit, University of Luxembourg, L-4422 Belvaux, Luxembourg.*

[+] equally contributing authors

* correspondence to: stephan.buecheler@empa.ch

## Supplementary Figures

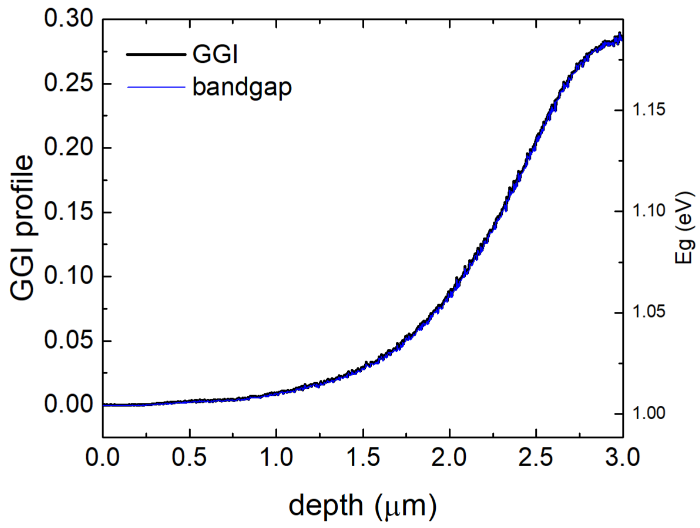


**Supplementary Figure 1 - GGI grading for the back graded bg-CIGS device as measured by SIMS (blue dots), see Ref. [2]. The grading is confined to the back contact, while the front contact still has a significant thickness of low bandgap CIS material for good absorption. The bandgap has been calculated as detailed in [3].**

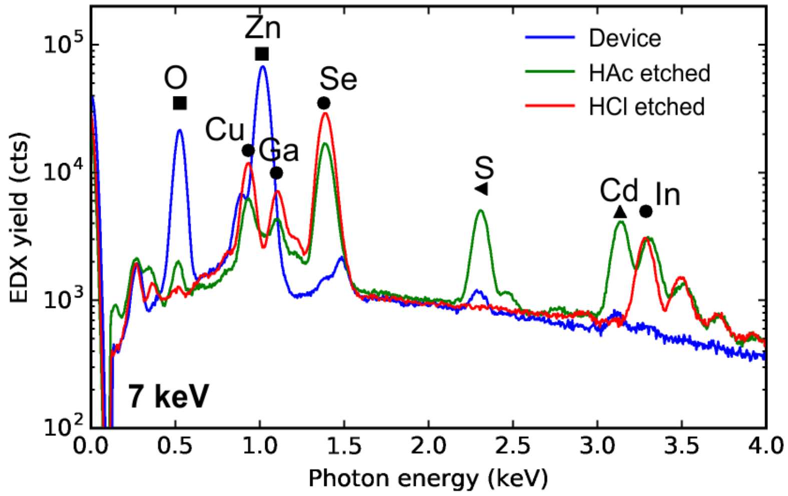


**Supplementary Figure 2 – EDX study of a full device (double graded CIGS absorber) (blue line) and after an etching in HAc (green line) or HCl (red line). The full device shows a clear signal for Zn and O. After the HAc etch these peaks vanish and the signals for Cd and S indicate that the CdS layer is still present. For an etch with HCl mainly the peaks for Cu, In, Ga and Se are present. An additional TRPL study on absorbers (double graded CIGS) directly after the CdS deposition and on devices after the HAc etch (removal of ZnO) show similar lifetimes (not shown).**

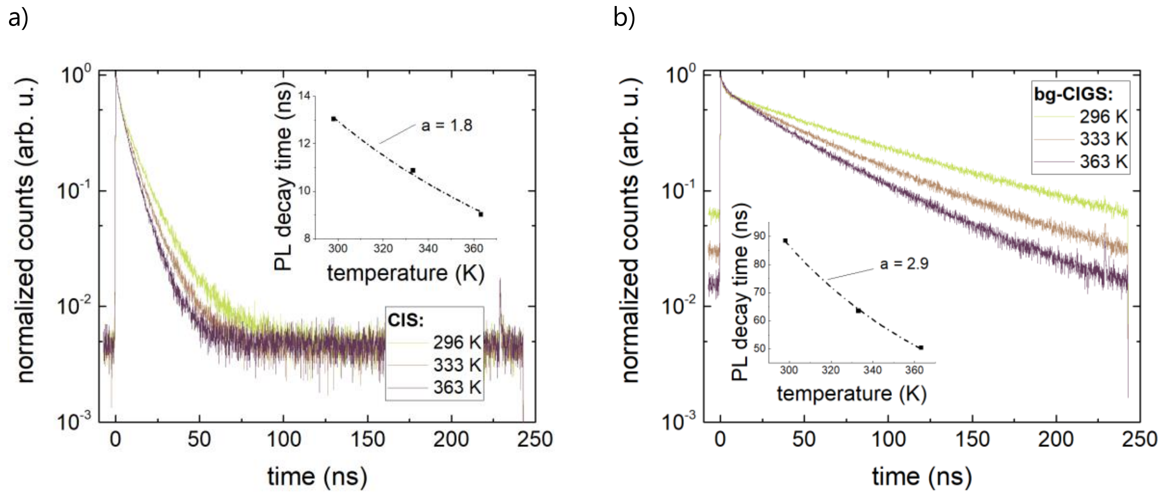


**Supplementary Figure 3 – Temperature dependence of the PL decay time for the CIS (a) and the bg-CIGS (b) absorber in the *glued* configuration. Lifetimes were extracted by fitting a single exponential function and are displayed in the respective insets. The temperature dependence was fitted using the expression $\tau(T) = \tau_0(T/300K)^{-a}$. An exponent of $a$ around 1.5 is expected for SRH recombination [4]. The slightly increased exponent of 2.9 for the bg-CIGS absorber might be explained by thermal emission of minority charge carriers over the back-grading [5].**

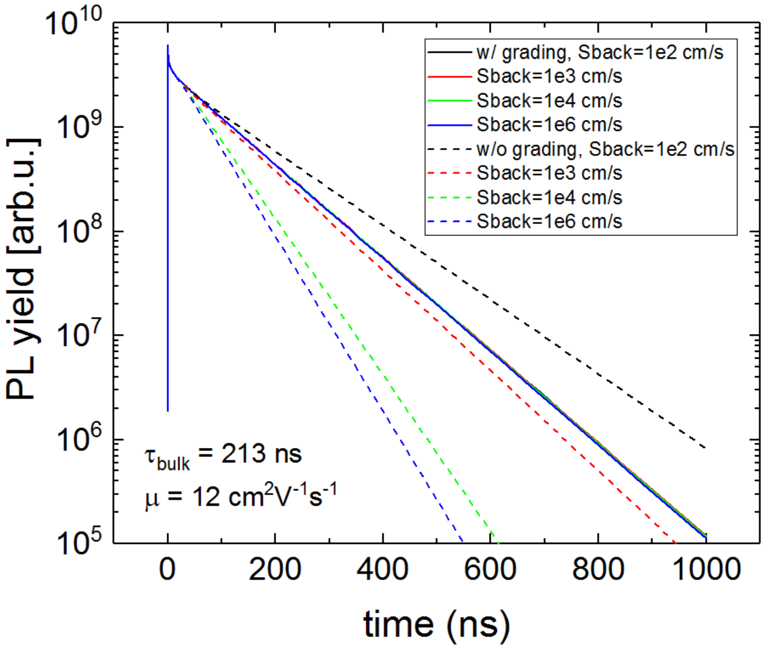


**Supplementary Figure 4 - Simulated PL transients w/ and w/o a conduction band grading towards the back contact. The thickness of the absorber was 3 $\mu m$ and the grading of the conduction band was linear from 1.5 $\mu m$ to the back contact with a total increase of 193 meV. For the graded sample (solid lines) the transients do not depend on the back surface recombination, which is in contrast to the transients w/o a back grading.**

# Supplementary Information A – Matlab script for TRPL transients

## Physical equations

In order to simulate the electron and hole densities as a function of time the Matlab script solves equations (E.1) through (E.8) simultaneously using the Matlab solver pdepe. Subsequently, the PL transient is calculated by integration of the radiative recombination over the whole absorber.

The transport equations for electrons and holes are [6,7]

$$\frac{\partial n}{\partial t} = k_B T \mu_n \frac{\partial^2 n}{\partial z^2} - \mu_n \frac{\partial}{\partial z}(nE_n) + \sum_i (e_{e,i} - e_{c,i}) - R_{rad} + G \tag{E.1}$$

$$\frac{\partial p}{\partial t} = k_B T \mu_p \frac{\partial^2 p}{\partial z^2} - \mu_p \frac{\partial}{\partial z}(pE_p) + \sum_i (h_{e,i} - h_{c,i}) - R_{rad} + G \tag{E.2}$$

The first term in equation (E.1) denotes the diffusion of electrons with $k_B$ the Boltzmann constant and $T$ the temperature. The second term represents the drift of the electrons in the presence of an electric field $E_n$ or more generally in the presence of an energy potential gradient. The grading of the conduction band due to the GGI grading is implemented by defining the electric field for the electrons $E_n = dE_C/dx$.

The third term describes electron emission $e_{e,i}$ and capture $e_{c,i}$ from and into a bandgap defect state with index $i$. Equation (E.2) is the analogous expression for holes. The defect state $i$ is characterized with total defect density $N_{t,i}$, energy distance from the conduction band $E_{t,i}$ and capture cross sections $\sigma_{n,i}$ and $\sigma_{p,i}$ for electrons and holes, respectively. The emission and capture rates in equation (E.1) and (E.2) can then be written as

$$e_{e,i} = n_{t,i}\sigma_{n,i}v_t N_C \exp\left(-\frac{E_{t,i}}{k_B T}\right) \tag{E.3}$$

$$e_{c,i} = n(N_{t,i} - n_{t,i})\sigma_{n,i}v_t \tag{E.4}$$

$$h_{c,i} = (N_{t,i} - n_{t,i})\sigma_{p,i}v_t N_V \exp\left(-\frac{E_g - E_{t,i}}{k_B T}\right) \tag{E.5}$$

$$h_{c,i} = p n_{t,i}\sigma_{p,i}v_t \tag{E.6}$$

The variable $n_{t,i}$ denotes the density of the defect state occupied by an electron. Furthermore, $N_c$ and $N_V$ denote the effective densities of states in the conduction and valence band, respectively and $v_t$ represents the thermal velocity. The dynamics of the defect state occupation density $n_{t,i}$ couples equations (E.1) and (E.2) and expresses as

$$\frac{\partial n_{t,i}}{\partial t} = -e_{e,i} + e_{c,i} + h_{e,i} - h_{c,i} \tag{E.7}$$

The fourth term in equation (E.1) describes the radiative recombination $R_{rad}$ as already described in equation (1). The last term in equation (E.1) denotes the optical generation of charge carriers and is described by equation (13).

The surface recombination velocities at the front ($S_{front,z=0}$) and the back ($S_{back,z=d}$) contact define boundary conditions for equations (E.1) and (E.2) and can be formalized according to [8,9]

$$k_B T \mu_n \left.\frac{\partial n}{\partial z}\right|_{z=0,d} + \mu_n n|_{z=0,d} E_n = -\left.\frac{np - n_0 p_0}{nS_{front,back}^{-1} + pS_{front,back}^{-1}}\right|_{z=0,d} \tag{E.8}$$

## Examples

This SI compiles comparison between published transients according to Refs. [1,4] and transients simulated at Empa using TCAD or the Matlab solver provided with this manuscript. Data from the references were extracted from the respective publications. The SI aims to confirm validity of solutions obtained with the Matlab solver for a range of relevant parameter variations. Here, only a brief description of the example output is given. More detailed description can be found in the example functions of the “TRPL_simulator_examples.m” script.

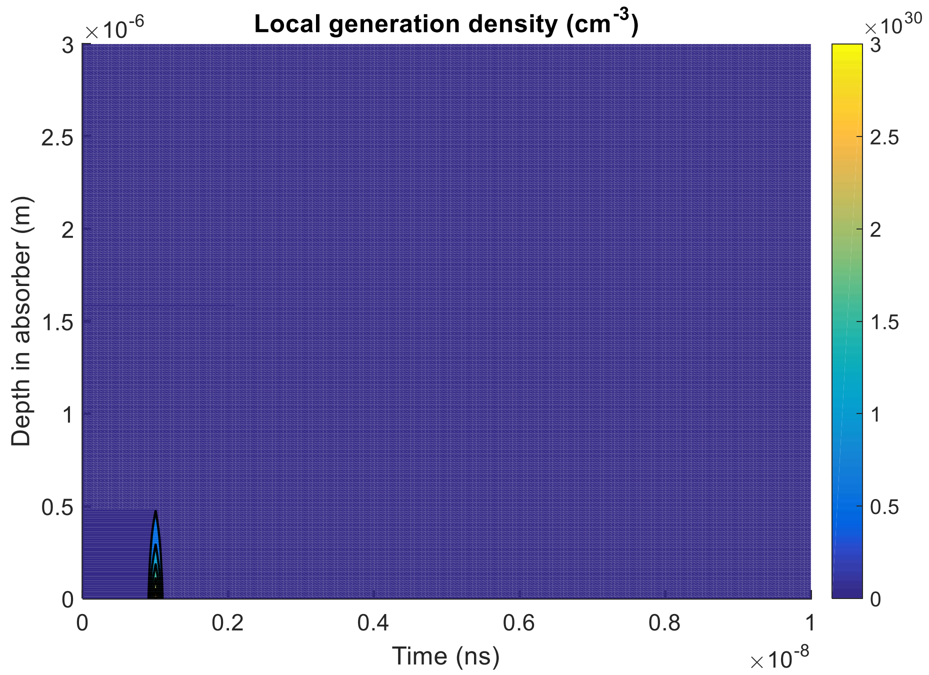


**Supplementary Figure 5 - The example shows how a gaussian type excitation pulse of a given width, at a given time and of a given excitation level can be created that can be passed to the TRPL solver. The output is a matrix over time and space containing excitation density at eacht point in time and space throughout the absorber layer and the simulation time. Shown here is a pulse of total excitation level of $10^{10}$ photons/pulse/cm$^2$ injected at 1 ns with a width of 100 ps into an absorber layer of 3 µm thickness and absorption coefficient of $3.806 \times 10^4$ cm$^{-1}$.**

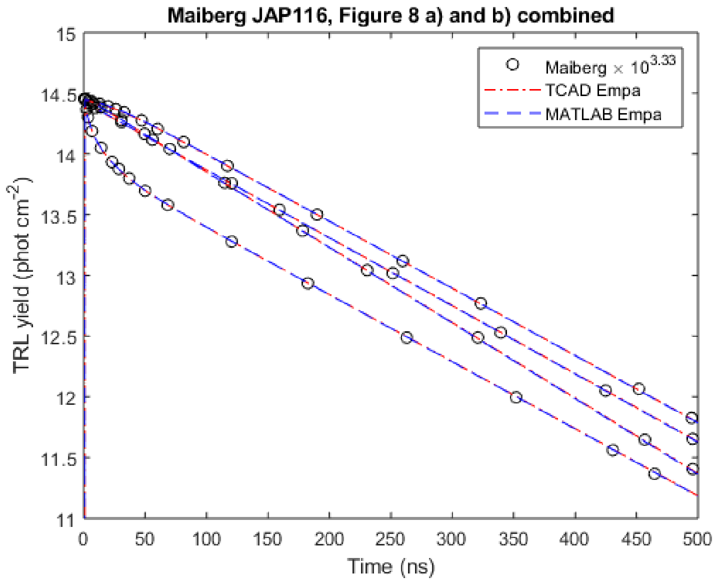


**Supplementary Figure 6 - The example reproduces transients from Maiberg et al. [1] Figures 8 a) and b) (combined) that feature a deep defect and different combinations of mobility and surface recombination velocities (see .m files or article). Empa TCAD as well as the Matlab solver give good reproduction of the transients presented in [1]. While for the Empa solvers the output is known to be in [photons/s/m$^2$] the transients from [1] here and in the following had to be scaled with a factor $10^{3.333}$ for an unknown reason.**

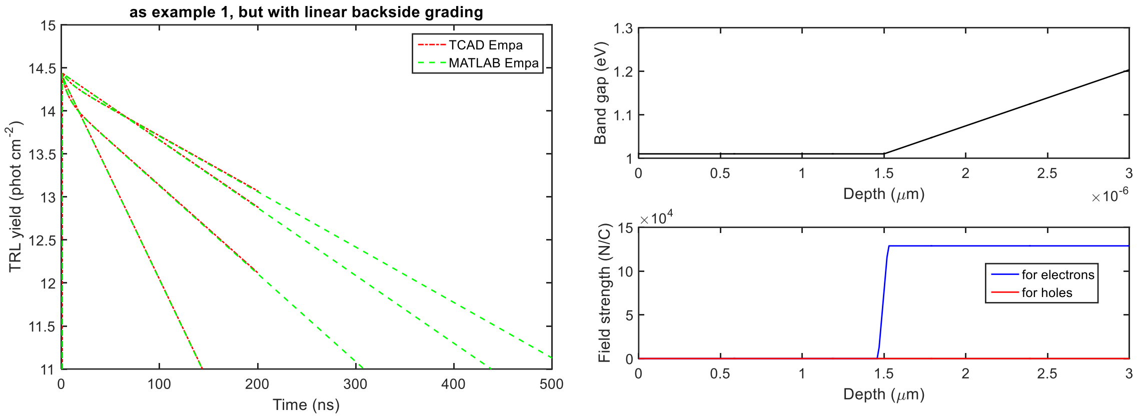


**Supplementary Figure 7 - a) The examples show simulations with same parameters as shown in Supplementary Figure 5, however a linearly increasing backside GGI grading was implemented. In Ref. [4] no such simulations are available therefore no Maiberg simulations can be shown but only Empa TCAD and Empa Matlab solutions are compared. b) GGI i.e. linear bandgap grading (top) as used for the simulations and the corresponding (generalized) electric fields (bottom) for electrons (blue) and holes (red). A simplified bowing function of $E_G$=1.01+0.69*GGI eV was used and the GGI increased from 0 towards 0.28 from 1.5 $\mu m$ towards 3 $\mu m$ absorber depth.**

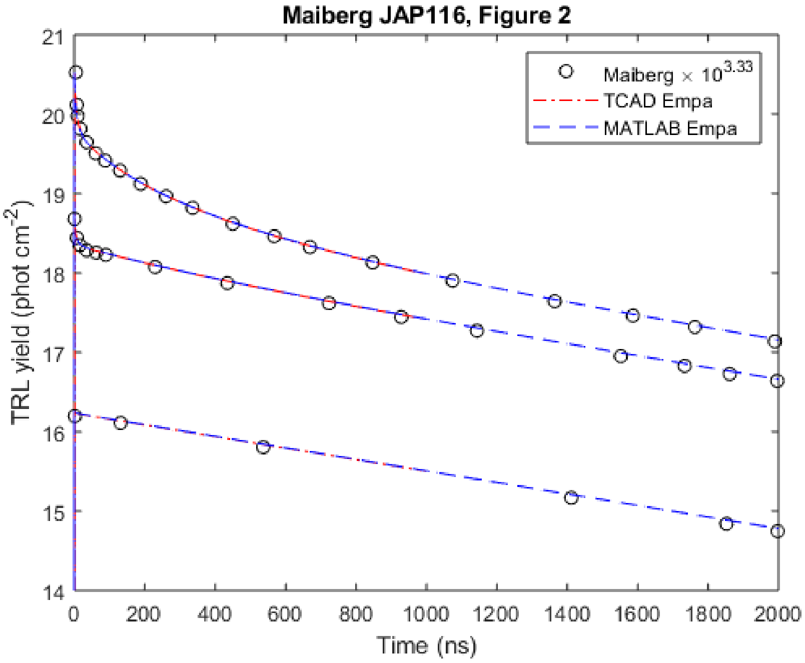


**Supplementary Figure 8 - Reproduces transients from Maiberg et al. [1] Figure 2. Bulk and surface recombination was disabled and only radiative recombination is active. Transients for three different excitation levels of $10^{13}$ cm$^{-2}$ pulse$^{-1}$, $10^{12}$ cm$^{-2}$ pulse$^{-1}$, $10^{10}$ cm$^{-2}$ pulse$^{-1}$ (from top to bottom) are shown.**

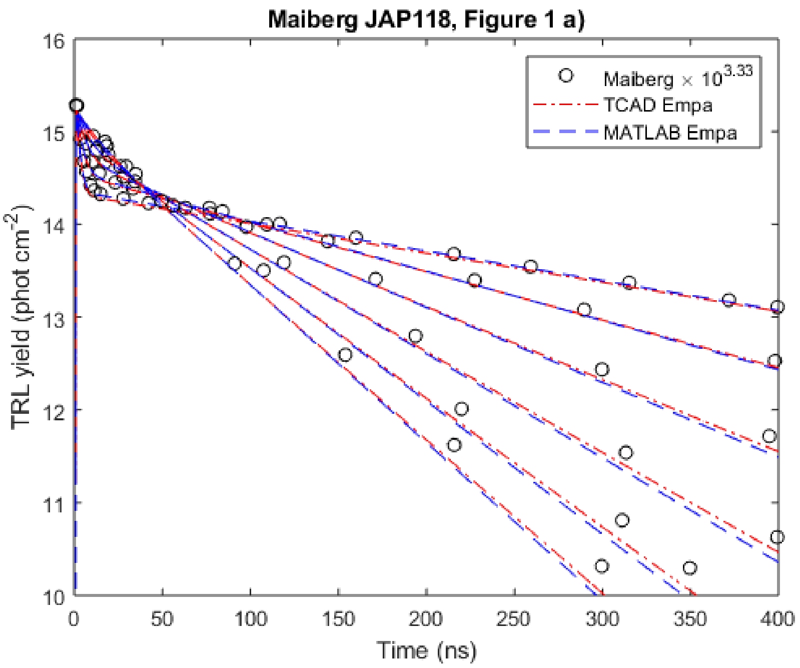


**Supplementary Figure 9 - Reproduces transients from Maiberg et al. [4] Figure 1. Parameters for mobility, surface and bulk recombination are fixed for all transients (see example function). One trap defect at 0.26 eV from the conduction band was introduced. The curves show a variation of the trap defect density, see example function for specific values. Notably, the explicit parameters that were varied in Ref. [4] were not given. It was found that a variation of the trap density can reproduce the transients presented in Ref. [4].**

# Supplementary Information B

1. Estimation of the doping density from capacitance voltage measurements

The doping density was measured by capacitance voltage (CV) measurements. The samples were kept in the dark at 50 °C for approximately 1h prior to the measurement to bring the sample in a relaxed state. Subsequently, CV curves were acquired from low to high temperatures at a frequency of 1 kHz and a level voltage of 30 mV. The doping density was then determined from a CV curve at a temperature, where the device is in the low frequency regime of an observed frequency dependent capacitance step as described in Ref. [10].

2. Choice for the back surface recombination velocity $S_{back,Mo}$ at the CIGS/Mo interface

In literature values for $S_{back,Mo}$ around $8 \times 10^4 cms^{-1}$ were estimated from modeling of electron beam induced current (EBIC) measurements [11,12]. Other reports on EBIC modeling used values in the range of $1 \times 10^5 cms^{-1}$ [13] to $1 \times 10^7 cms^{-1}$ [14] however without discussion of this choice. Also a dependency of the CIGS/Mo interface recombination velocity on the interface Na content was suggested [15]. The estimates are based on a TRPL study of ultrathin (< 200 nm) CIGS absorber layers that underwent different durations of NaF PDT to introduce different amounts of Na. The Na concentration across the layers was profiled by means of SIMS. The authors reported that $S_{back,Mo}$ ranges from $1 \times 10^2 cms^{-1}$ (for the highest amount of Na) to $>1 \times 10^5 cms^{-1}$ (for lower Na amounts). A direct comparison of the SIMS measurements to other samples discussed in this study is hindered by the non-absolute nature of the SIMS technique. Also, a possible effect of forward sputtering that could affect the Na count at the rear interface (sputtering from front towards back surface) was not discussed.

In addition, in this study TRPL measurements were also performed from the rear side of the CIS layer through a 6 nm Au layer (*delam* configuration with e-beam evaporated Au layer on the exposed interface, not shown here). In that case the luminescence decreased extremely fast with decay times in the range of 1 ns indicative for a large recombination velocity at the CIS/Au interface. However, measurements from the front side show similar decay times for the CIS/Mo (*glued* configuration for CIS absorber) and the CIS/Au (not shown) configuration indicating a similar and consequently a rather large interface recombination velocity for the CIS/Mo (and CIS/Au) interface.

# Bibliography


1 Maiberg, M. & Scheer, R. Theoretical study of time-resolved luminescence in semiconductors. II. Pulsed excitation. *Journal of Applied Physics* 116, doi:10.1063/1.4896484 (2014).

2 Feurer, T. *et al.* Single-graded CIGS with narrow bandgap for tandem solar cells. *Science and Technology of Advanced Materials* 19, 263-270, doi:10.1080/14686996.2018.1444317 (2018).

3 Carron, R. *et al.* Refractive indices of layers and optical simulations of $Cu(In,Ga)Se_2$ solar cells. *Science and Technology of Advanced Materials* 19, 396 - 410, doi:10.1080/14686996.2018.1458579 (2018).

4 Maiberg, M., Hölscher, T., Zahedi-Azad, S. & Scheer, R. Theoretical study of time-resolved luminescence in semiconductors. III. Trap states in the band gap. *Journal of Applied Physics* 118, doi:10.1063/1.4929877 (2015).

5 Weiss, T. P. *et al.* Time-resolved photoluminescence on double graded Cu(In,Ga)Se2 – impact of front surface recombination and its temperature dependence. ((submitted)).

6 Sze, S. & Ng, K. K. *Physics of Semiconductor Devices: Third Edition*. (2006).

7 Neamen, D. *Semiconductor physics and devices*. (McGraw-Hill, Inc., 2002).

8 Hsu, W. W. *et al.* Surface passivation of Cu(In,Ga)Se 2 using atomic layer deposited Al 2O 3. *Applied Physics Letters* 100, doi:10.1063/1.3675849 (2012).

9 Aberle, A. G., Glunz, S. & Warta, W. Impact of illumination level and oxide parameters on Shockley-Read-Hall recombination at the Si-SiO2 interface. *Journal of Applied Physics* 71, 4422-4431, doi:10.1063/1.350782 (1992).

10 Weiss, T. P. *et al.* Impact of annealing on electrical properties of Cu2ZnSnSe4 absorber layers. *Journal of Applied Physics* 120, doi:10.1063/1.4959611 (2016).

11 Nichterwitz, M. *et al.* Influence of grain boundaries on current collection in Cu(In,Ga)Se2 thin-film solar cells. *Thin Solid Films* 517 (2009).

12 Kavalakkatt, J. *et al.* Electron-beam-induced current at absorber back surfaces of Cu(In,Ga)Se 2 thin-film solar cells. *Journal of Applied Physics* 115, doi:10.1063/1.4858393 (2014).

13 Brown, G. *et al.* Determination of the minority carrier diffusion length in compositionally graded Cu (In,Ga) Se2 solar cells using electron beam induced current. *Applied Physics Letters* 96, doi:10.1063/1.3291046 (2010).

14 Nichterwitz, M. & Unold, T. Numerical simulation of cross section electron-beam induced current in thin-film solar-cells for low and high injection conditions. *Journal of Applied Physics* 114, 134504 (2013).

15 Jarzembowski, E. *et al.* The influence of sodium on the molybdenum/Cu(In,Ga)Se<inf>2</inf> interface recombination velocity, determined by time resolved photoluminescence. *Applied Physics Letters* 107, doi:10.1063/1.4928187 (2015).
